# Brief Postnatal Visual Deprivation Triggers Long-Lasting Interactive Structural and Functional Reorganization of the Human Cortex

**DOI:** 10.3389/fmed.2021.752021

**Published:** 2021-11-17

**Authors:** Yixuan Feng, Olivier Collignon, Daphne Maurer, Ke Yao, Xiaoqing Gao

**Affiliations:** ^1^Eye Center of the Second Affiliated Hospital, School of Medicine, Zhejiang University, Hangzhou, China; ^2^Zhejiang Provincial Key Lab of Ophthalmology, Hangzhou, China; ^3^Institute of Research in Psychology/Institute of Neuroscience, University of Louvain, Louvain-la-Neuve, Belgium; ^4^Centro Interdipartimentale Mente/Cervello, Università di Trento, Trento, Italy; ^5^Department of Psychology, Neuroscience & Behaviour, McMaster University, Hamilton, ON, Canada; ^6^The Hospital for Sick Children, Toronto, ON, Canada; ^7^Center for Psychological Sciences, Zhejiang University, Hangzhou, China

**Keywords:** congenital cataracts, early visual deprivation, cortical thickness covariation, functional connectivity, magnetic resonance imaging

## Abstract

Patients treated for bilateral congenital cataracts provide a unique model to test the role of early visual input in shaping the development of the human cortex. Previous studies showed that brief early visual deprivation triggers long-lasting changes in the human visual cortex. However, it remains unknown if such changes interact with the development of other parts of the cortex. With high-resolution structural and resting-state fMRI images, we found changes in cortical thickness within, but not limited to, the visual cortex in adult patients, who experienced transient visual deprivation early in life as a result of congenital cataracts. Importantly, the covariation of cortical thickness across regions was also altered in the patients. The areas with altered cortical thickness in patients also showed differences in functional connectivity between patients and normally sighted controls. Together, the current findings suggest an impact of early visual deprivation on the interactive development of the human cortex.

## Introduction

The human cortex takes many years to develop an adult-like structure, with major postnatal changes in volume, surface area, cortical thickness, myelination, and connectivity (1). Changes in cortical thickness are particularly interesting because they likely reflect developing patterns of neuronal connectivity supporting the functional specialization of brain networks (1–5). In most cortical areas, cortical thickness increases during the first year, followed by stabilization in the second year (6, 7). By age three-four, a monotonic decrease in cortical thickness that continues until at least age 30 is observed (1, 8–13). These patterns are seen throughout the brain, including the occipital lobe, which undergoes small increases in thickness during the first year (6, 7), and later monotonic reductions. The developmental thinning has been attributed to experience-dependent pruning (14). although contributions from changes in cell size, cortical stretching, and/or sulci width or depth cannot be ruled out (8, 9).

The role that sensory experience plays in shaping the development of the structure of brain networks remains elusive. In this context, the study of people born with atypical sensory experience represents a unique opportunity to probe how changes in sensory experience influence the development of brain structures (15). Evidence of the role of early visual experience in cortical thickness comes from studies contrasting the early blind to those whose blindness started later in childhood. In adults who became blind at an early age, the occipital cortex is thicker than normal (16, 17). Although the exact areas where enhanced thickness is observed vary from study to study, they usually include pericalcarine regions (18–20), and often the lingual gyrus (20, 21). Other areas of enhanced cortical thickness that have been reported in adults with early onset of blindness include the cuneus (18), the lateral occipital cortex (17, 18, 21), the right rostral middle frontal gyrus (20), and the left caudal anterior cingulate gyrus (20). No such changes are seen in those whose blindness started later in childhood, even when blindness began as early as 2 years of age (16, 20, 21). The contrasting results for the early blind vs. those blind later in childhood indicate an influence of visual experience during infancy in shaping the cortical thinning documented from about age three in normal development.

Enhanced cortical thickness in the early blind is usually attributed to reduced pruning of exuberant synapses based on visual experience (16, 20): Because visual experience cannot reinforce some synapses more than others, there is no basis for selective enhancement of those that are often engaged and selective pruning of those that are not stimulated. Instead, the spontaneous activity will have an unselective effect, perhaps punctuated by effective stimulation of some neurons by other sensory modalities through cortico-cortical connections (22, 23). In visually normal infants, such activation of the occipital cortex is minor compared to the dominant structured visual stimulation and hence most of the direct connections among primary sensory cortices are pruned away (18).

This interpretation is consistent with evidence that the amount of auditory activation in the occipital cortex of early blind is negatively correlated with the thickness in the calcarine sulcus, cuneus, lingual gyrus, and superior occipital gyrus (18). In other words, when input from the ectopic sensory modality is effective in strengthening connections to the visual cortex (24, 25) a pruning process is implemented that thins that cortex, presumably by eliminating the many synapses that are weakly activated. Recent work with the early blind suggested that the influence of early experience on the development of brain structure goes beyond the visual cortex, as cortical thickness in occipital areas is less strongly correlated with thickness across many brain areas, including the frontal regions (26). Consistent with that idea is Hasson et al.'s findings (27) from graph-theoretic metrics, that the whole-brain networks of regional cortical thickness covariation in the early blind differed from that found for the sighted. However, aside from the indirect evidence from contrasting the early blind with the late blind or the sighted (28), there is no direct evidence elucidating the role of early visual experience on shaping the structure of the occipital cortex and its connectivity with other cortical areas.

Studies of children born with dense bilateral cataracts afford an opportunity to directly examine the specific role of early visual experience on cortical development. Unlike the early blind to whom the visual deprivation persists, the deprivation is restricted to an early period of development because the congenital cataract patients had the cataractous lenses removed surgically during infancy and the eyes fitted with compensatory contact lenses to supply (nearly) normal visual input. The cataract-reversal patients later have defects in a number of aspects of vision, e.g., acuity, contrast sensitivity, visual form and motion (29–31), face and object perception (32–35), enhanced auditory processing (36, 37), and abnormal audio-visual integration (34, 37–39), although some remain unaffected (28, 40). In addition, neuroimaging data show that auditory stimuli activate visual cortical regions in the cataract-reversal patients (36). Collectively, these behavioral and neuroimaging data suggest altered intersensory connections may contribute to the visual deficits (23). In the only study to date on cortical thickness, Guerreiro and colleagues compared six adults with a history of early visual deprivation from cataracts between birth and 5–25 months of age to six normally sighted adults (41). An ROI-based analysis revealed enhanced cortical thickness in the cataract-reversal group in the calcarine sulcus (on the left) and bilaterally in the superior occipital gyrus and transverse occipital gyrus. The authors suggested the results imply that the absence of early visual input putatively reduces visually-driven pruning of unused synapses (14). However, likely limited by a small sample size, this previous study did not compare cortical thickness between patients and controls at the whole-brain level or look at cortical thickness covariance across brain regions.

In the current study, we evaluated the role of early visual input in shaping the structure of the occipital cortex and its connectivity with other cortical areas in a larger sample (*n* = 11) of adults treated for bilateral congenital cataracts, all of whose visual deprivation was restricted to a relatively short period (<9 months). This gave us a better opportunity to reveal the influence of early visual experience on the processes underlying cortical thinning beyond the visual cortex. Specifically, we looked for differences in cortical thickness across the whole brain, as well as regional cortical-thickness covariance. To do so, first, we determined the regions of cortical thickness altered in the cataract-reversal group and the correlations among them. Second, we examined the interrelationships in cortical thickness in the whole brain using the left calcarine sulcus as a seed. This area was identified as thicker in the current sample of cataract-reversal patients and in the only previous study of such patients, and in most studies of the early blind. Based upon the results of the cortical thickness analyses, we assessed resting-state functional connectivity (42) in areas showing altered cortical thickness in order to investigate potential changes in functional connectivity that coincides with changes in cortical thickness.

## Materials and Methods

### Participants

We tested 11 adult patients who had been diagnosed by an ophthalmologist with dense central bilateral congenital cataracts that prevented any patterned visual input until the cataracts were removed surgically and the eyes fit with compensatory contact lenses. The duration of visual deprivation varied between 65 and 283 days (mean = 138 days, based on the eye with shorter deprivation if it differed between the two eyes). On the day of testing, the corrected visual acuity of the better eye ranged from 20/20 to 20/125 (median = 20/32). We tested 24 age- and sex-matched control participants (requiring that for each patient there was at least one sex-matched control participant within ± 2 years of age) for the cortical thickness analysis and 15 age- and sex-matched control participants (with the same age matching requirement) for the comparison of resting-state functional connectivity analysis. All control participants reported no history of visual abnormality and had normal or corrected-to-normal vision (20/20 or better in each eye). All patients and control participants were right-handed. None of them reported any history of psychiatric or neurological disorders or current use of any psychoactive medications. All procedures were in accordance with the 1964 Helsinki declaration and its later amendments or comparable ethical standards. The research protocols were approved by the research ethics committees of McMaster University, The Hospital for Sick Children in Toronto, and York University. We collected informed written consent from each participant.

### MRI Acquisition

We acquired anatomical and resting-state functional magnetic resonance imaging (RS-fMRI) images using a three-Telsa Siemens Magnetom Trio system (Siemens Medical System, Erlangen, Germany) with a 32-channel head coil. The anatomic images were collected using a high-resolution T1-weighted magnetization-prepared gradient-echo image (MP-RAGE) sequence [192 sagittal slices, repetition time (TR) = 2,300 ms, echo time (TE) = 2.62 ms, voxel size = 1 mm isotropic, flip angle (FA) = 9°, field-of-view (FoV) = 256 × 256 mm^2^, matrix size = 256 × 256, parallel scanning mode = GRAPPA, accelerate factor = 2]. The acquisition time for the anatomic scan was 321 seconds. Resting-state functional images were collected using a T2^*^ weighted gradient-echo (GE) echo planar imaging (EPI) sequence (37 axial slices, interleaved, slice thickness = 3.5 mm with no gap, TR = 2,250 ms, TE = 30 ms, voxel size = 3.0 × 3.0 × 3.5 mm^3^, flip angle = 90°, FoV = 240 × 240 mm^2^, matrix size = 80 × 80). The functional scan took 540 s and acquired 240 whole-brain volumes.

### Data Preprocessing

The anatomical MRI data were analyzed with Freesurfer (version 5.3.0, http://surfer.nmr.mgh.harvard.edu, running on Mac OSX 10.10.5). We reconstructed a cortical surface model from each T1 weighted image using the automated processing pipeline in Freesurfer with default parameters. This fully automated process has been described previously in detail (43, 44). Briefly, the major components include Talairach transformation, intensity normalization, removal of non-brain tissue, segmentation and tessellation of the gray and white matter boundary, and automatic topology correction. We visually inspected the reconstructed cortical surfaces for errors that might have occurred during the brain extraction and segmentation procedure and performed manual editing to fix the errors when necessary.

The RS-fMRI data were pre-processed using Data Processing and Analysis for Brain Imaging (DPABI V4.2, http://www.rfmri.org/dpabi) (45) and Statistical Parametric Mapping (SPM12, https://www.fil.ion.ucl.ac.uk/spm/software/spm12/) with MATLAB (Release 2014a, https://www.mathworks.com/). After removing the first 10 volumes and slice-timing correction, the functional images were realigned to the first image for motion correction. A criterion of maximum translation exceeding 2.5 mm or rotation exceeding 2.5° did not detect any participant with excessive movement. For each participant, the 3D anatomical image was co-registered to the time-averaged functional image, spatially segmented by “Dartel + Segment,” and resampled into 3 × 3 × 3 mm^3^ voxels. Then, we removed temporal linear trend from the functional time series data, performed regression of movement parameters from motion correction with the Friston-24 parameter model, and performed regressions of white matter (WM), cerebrospinal fluid (CSF), and global mean signal on the time series data. All RS-fMRI images were spatially normalized into the standard Montreal Neurological Institute (MNI) brain space using Dartel and then smoothed with a 6 mm Gaussian kernel.

### Cortical Thickness Measurements

From the resulting cortical surface, cortical thickness was calculated at each vertex. Each individual brain surface was registered to a common spherical atlas to allow inter-subject averaging and statistical analysis. The reconstructed cortical surface was automatically parcellated into gyri-based regions based on cortical folding patterns (46).

### Statistical Analysis

We evaluated the regional differences in cortical thickness between the patients and the controls using a vertex-by-vertex general linear model (GLM) using FreeSurfer's Qdec (version 1.4) with group (patients vs. controls) and gender as fixed effects and age as covariate. Before the statistical evaluation, cortical thickness maps were smoothed with a surface-based Gaussian kernel (full-width-half-maximum = 10 mm) to reduce the effect of misalignment when registering each individual cortical surface to the common spherical template. We identified significant vertex clusters using a threshold of *P* < 0.001 (uncorrected)^1^ and a minimum cluster size of 30 mm^2^.

For resting-state functional connectivity analysis, we first computed regional mean time series for each participant by averaging the fMRI time series over six spheric regions of interest (ROIs) (radius = 3 mm) centered at the peak voxel from vertex clusters differing significantly between patients and controls in cortical thickness. We then calculated the functional connectivity between each ROI and all voxels in the whole brain by Pearson's correlation to obtain subject-specific functional connectivity maps. These functional connectivity maps were standardized by Fisher's z-transformation so that individual maps could be averaged and compared among participants. For each ROI, we performed one-sample *t*-tests in the patients and in the controls to identify brain regions with significant connectivity with each ROI. Then within the regions showing significant connectivity with each ROI, a two-sample *t*-test was performed between the patients and controls to identify areas showing different connectivity between patients and controls. We corrected for multiple comparisons using random field theory (47) (voxel level *P* < 0.001, cluster level *P* < 0.05).

## Results

### Altered Cortical Thickness Beyond Visual Cortex in Cataract-Reversal Patients

**Figure 1:**
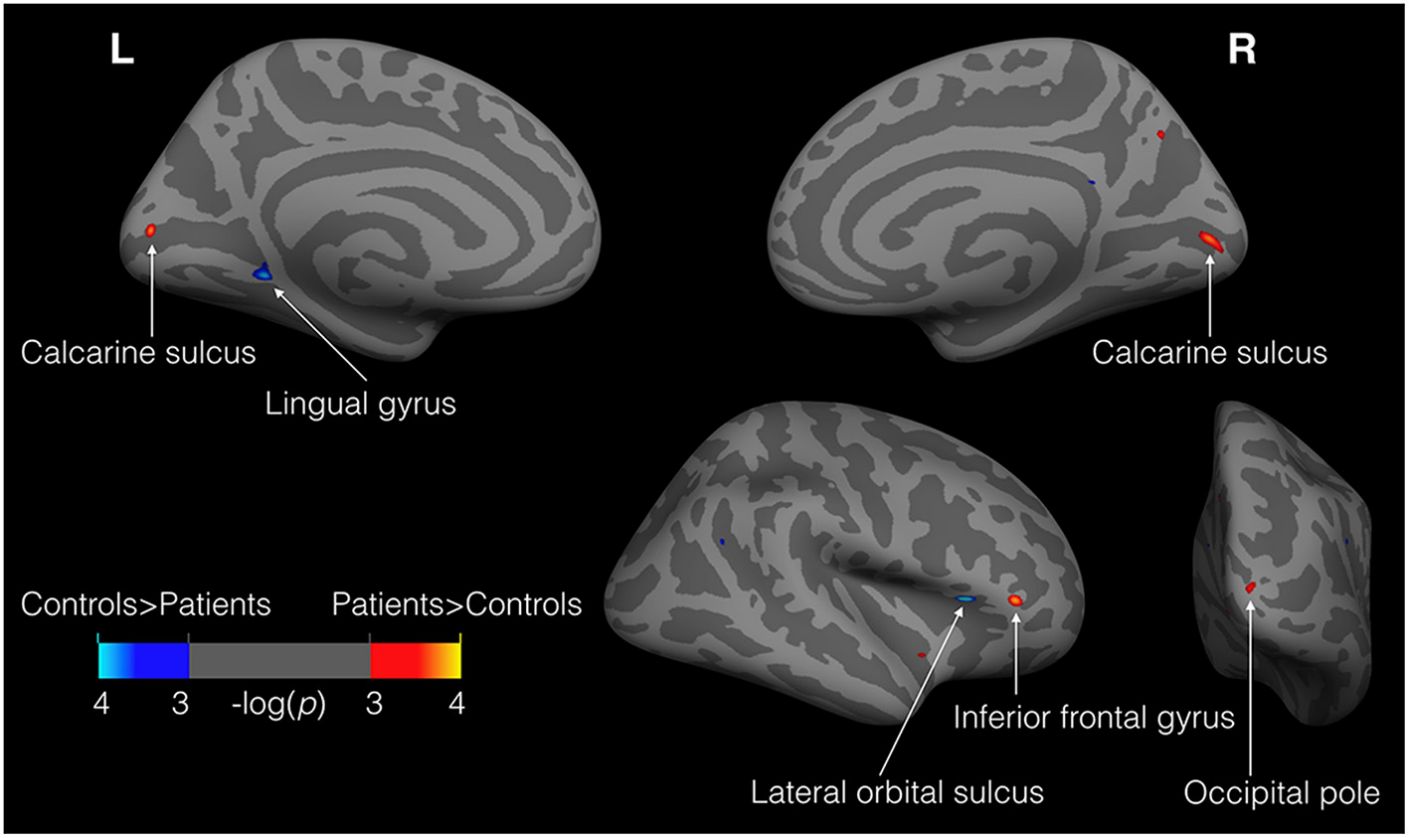
Loci showing differences in cortical thickness between patients and controls (11 patients vs. 24 controls). Illustrated here are the results from GLM analysis on cortical thickness with group (patients vs. controls) and gender as fixed effects and age as covariate. Warm colors (red to yellow) represent areas where patients have thicker cortex than the controls, while cold colors (blue to cyan) represent the reverse. The maps were thresholded at *P* < 0.001 (uncorrected). Only clusters >30 mm^2^ are labeled.

A whole-brain vertex-by-vertex analysis revealed differences in regional cortical thickness between cataract-reversal patients and controls in areas that go well-beyond the visual cortex. Table 1 summarizes the cluster size, mean cortical thickness, and statistical significance in the comparison between patients and controls. As shown in Figure 1, the cataract-reversal patients have thicker cortex than the controls in the calcarine sulcus in both hemispheres. Thicker cortex is also present in the occipital pole and the inferior frontal gyrus in the right hemisphere. At the same time, patients have thinner cortex than the controls in the left lingual gyrus and the right lateral orbital sulcus than the controls.

**Table 1:** Areas differing in cortical thickness between patients and controls.

**Area**	**Peak MNI (x,y,z)**	**Size (mm^**2**^)**	***P*-value**	**Patient mean (sd)**	**Control mean (sd)**
**Left hemisphere**					
Lingual gyrus	−35, −43, −14	74.5	0.00013	2.27 (0.33)	2.76 (0.38)
Calcarine sulcus	−7, −85, 20	40	0.00025	1.64 (0.10)	1.48 (0.14)
**Right hemisphere**					
Calcarine sulcus	19, −83, 13	70	0.00025	1.74 (0.23)	1.58 (0.11)
Inferior frontal gyrus	54, 36, −7	48.6	0.0002	2.89 (0.20)	2.58 (0.23)
Lateral orbital sulcus	46, 26, 1	32.3	0.00003	2.37 (0.17)	2.68 (0.22)
Occipital pole	19, −98, 22	34.8	0.00063	2.41 (0.18)	2.03 (0.31)

*Peak MNI coordinates, size and differences (P-values) between groups, as well as mean (standard deviation) cortical thickness at the peak of each area showing difference between patients and controls. MNI, Montreal Neurological Institute; sd, standard deviation*.

### Changes in Cortical Thickness Covariation

To test the interplay among areas that differ in cortical thickness between patients and controls, we calculated Pearson correlations among all pairs of areas listed in Table 1 for the patient group. We then calculated the 99% confidence intervals of the corresponding correlations in the data from the controls through bootstrapping (1,000 iterations). Specifically, for each bootstrap iteration, we randomly sampled data from 11 control participants (the same number as the patients) from a total of 24 control participants, with replacement. The 99% confidence intervals (CI) of the correlations calculated from the data of the controls served as baselines to determine if any correlation values of the patients deviate significantly from those of the controls. Unlike in controls, the thickness of the left Calcarine sulcus was negatively correlated with the thickness of the left Lingual gyrus (*r* = −0.71, *P* = 0.014, 99% CI of the control: −0.68 to 0.58). The thickness of the left Lingual gyrus was also negatively correlated with the thickness of the contralateral Calcarine sulcus (*r* = −0.67, *P* = 0.024, 99% CI of the control: −0.63 to 0.78). In addition, the thickness of the left Lingual gyrus was positively correlated with the thickness of the right inferior frontal gyrus (*r* = 0.71, *P* = 0.014, 99% CI of the control: −0.72 to 0.66). The increased magnitude of covariation in these areas in the patient group suggests interplay among cortical areas as a result of early visual deprivation.

**Figure 2:**
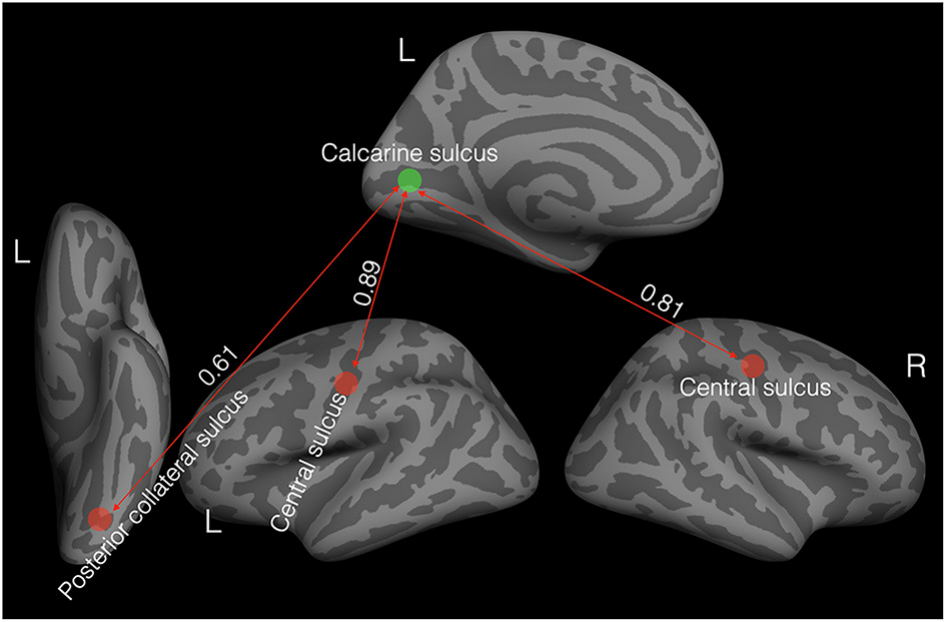
Elevated covariation in cortical thickness in the patients (*N* = 11). The green disc indicates the seed area: the left Calcarine sulcus. The red discs represent areas with positive correlations in cortical thickness with the seed area. All the correlation values are significantly different from zero and significantly higher than the corresponding correlation values in the controls based on bootstrapped 99% confidence intervals.

To further investigate such interplay in cortical thickness in the whole brain, we chose a seed area of theoretical significance. Specifically, we chose the left Calcarine sulcus, as it is found to be thicker in the current study and the one previous study (41) of cataract-reversal patients, and in most studies (16–21) of the early blind. We correlated the thickness of the seed area to the average thickness of 74 areas parcellated by freesurfer in each hemisphere (46). We accessed the significance of the correlation values of the patients in comparison to the 99% confidence interval of the corresponding bootstrapped correlation values of the controls. Figure 2 shows the areas where the correlations with the seed area in the patients deviate significantly from the corresponding correlations of the controls.

Unlike in the controls, the thickness of the left Calcarine sulcus was positively correlated with the thickness in bilateral central sulci (left: *r* = 0.89, *P* < 0.001, 99% CI of the controls: −0.56 to 0.79; right: *r* = 0.81, *P* = 0.002, 99% CI of the controls: −0.56 to 0.79) and the left posterior collateral sulcus (*r* = 0.61, *P* = 0.045, 99% CI of the controls: −0.83 to 0.45).

### Areas With Altered Cortical Thickness Also Showed Changes in Resting-State Functional Connectivity

**Figure 3:**
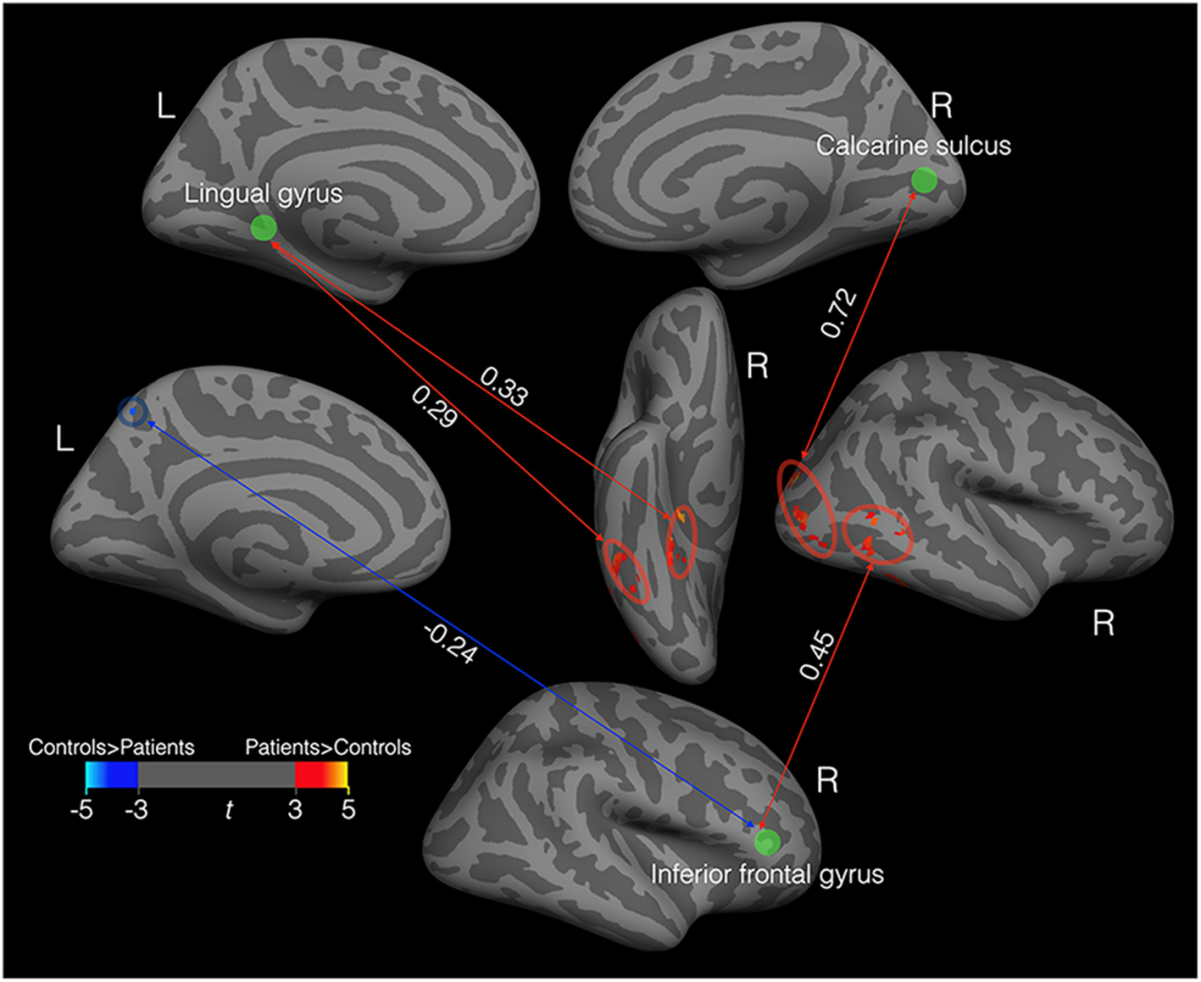
Functional connectivity in the patients (*N* = 11) that deviated from that in controls. The green discs indicate three spheric ROIs centered on the peak voxels of vertex clusters exhibiting significant differences in cortical thickness between patients and controls. The red ovals enclosed areas with stronger positive connectivity in patients than in the controls. The blue circle enclosed an area with stronger negative connectivity in patients than in the controls. All the correlation values (the white numbers) are significantly different from zero and significantly deviate from the corresponding correlation values in the controls.

We defined six spheric ROIs centered on the peak voxels of vertex clusters (Figure 1) exhibiting significant differences in cortical thickness between patients and controls. We then calculated resting-state functional connectivity between each ROI and all the voxels in the whole brain. Figure 3 shows pairs of brain areas (one in each pair is an ROI) showing a significant correlation in the patient group where the magnitude of correlation deviated significantly from that of the controls (voxel level *P* < 0.001, cluster level *P* < 0.05, corrected using random field theory, *n* = 26, degree of freedom = 22). Compared to the controls, the patients showed stronger positive functional connectivity between the left lingual gyrus and the right ventral temporal cortex including the lateral fusiform gyrus and the parahippocampal sulcus. The right calcarine ROI in the patients showed stronger positive functional connectivity with the right lateral occipital area. The right inferior frontal gyrus ROI in the patients showed stronger positive functional connectivity with the right middle temporal gyrus and stronger negative functional connectivity with the left precuneus area.

### Effects of Duration of Deprivation and Visual Acuity on Cortical Thickness Development

**Figure 4:**
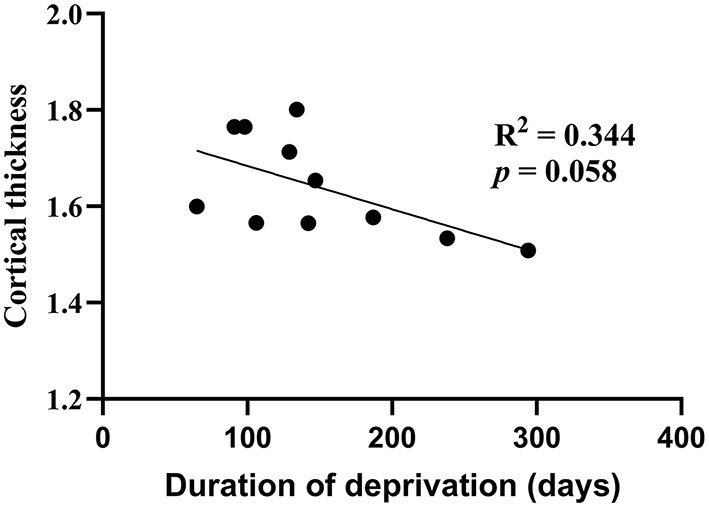
Correlation of cortical thickness (mm) between the left calcarine sulcus and duration of deprivation (days) in cataract-reversal patients (*N* = 11).

The covariation in cortical thickness between the left calcarine sulcus and other areas of the brain highlights the importance of early visual experience in the interactive development of the cortex. We tested if the thickness of the left calcarine sulcus is directly associated with the duration of early visual deprivation and visual acuity. Within the current sample of cataract-reversal patients, there is no correlation between the visual acuity and cortical thickness in the left Calcarine sulcus (*r* = 0.37, *P* = 0.26). However, there is a trend for cortical thickness in the left calcarine sulcus to be negatively correlated with the duration of deprivation (*r* = −0.587, *P* = 0.058, Figure 4; Such a trend should be interpreted with caution given the small sample size, as it may be affected by single data points. If the data point on the bottom right was removed, the trend would disappear, *r* = −0.449, *P* = 0.193). In addition, within the patients, cortical thickness in none of the other areas showing difference in cortical thickness between patients and controls is correlated to either visual acuity or duration of deprivation.

## Discussion

Adults treated for bilateral congenital cataracts provide a unique opportunity to study the effect of a short and transient period of early visual deprivation on the development of the visual cortex. Here, in a group of adult cataract-reversal patients who were deprived of any patterned visual input from birth for a period of 2–9 months, we found increased cortical thickness in the occipital cortex including the calcarine sulcus in both hemispheres, where the primary visual cortex (V1) is concentrated, and the occipital pole in the right hemisphere. The findings are consistent with the only previous study (41) on cataract-reversal patients, which reported increased cortical thickness in multiple ROIs in the occipital cortex including the left calcarine sulcus in a sample with longer average deprivation (5–24 months). Thus, our study confirms and extends those results by showing that visual experience in the first few months of life impacts the process of cortical thickening and thinning in the occipital lobe.

Our study also revealed that there are differences in the right calcarine sulcus, consistent with evidence of increased cortical thickness in bilateral calcarine sulcus in the early blind (16–21). Our whole-brain analysis revealed for the first time that there is also increased cortical thickness in the inferior frontal gyrus in the cataract-reversal patients. Thus, the current results indicate that the effect of early visual deprivation on the development of the cortex is not limited to the visual cortices, a result similar to what has been found with the early blind (20, 21).

Besides areas that showed increased cortical thickness in comparison to the normally sighted controls, our results reveal for the first time that there is decreased cortical thickness in the lingual gyrus (left) and the lateral orbital sulcus (right) in the cataract-reversal patients relative to the controls. Interestingly, among the cataract-reversal patients, the cortical thickness in the lingual gyrus is correlated negatively with the cortical thickness in bilateral calcarine. Such negative correlations in cortical thickness between different cortical areas in the cataract-reversal patients suggest an interaction among cortical regions driven by early visual input. Thus, it provides support to the interactive specialization view (48) from a sensory deprivation perspective.

Such interplay between the cortical thickness among different areas was confirmed by correlations between a seed area (the left calcarine sulcus) and the average thickness of anatomically defined cortical areas in the rest of the brain. In comparison to the normally sighted controls, the patients showed elevated covariation between the thickness of the left calcarine sulcus and the thickness of the posterior collateral sulcus on the ventral surface of the left temporal lobe and bilateral central sulci. To our knowledge, the current study is the first to demonstrate that a brief period of early visual deprivation can affect cortical thickness covariation at the whole-brain level.

Among the six loci that showed altered cortical thickness in the cataract-reversal patients, three also showed altered resting-state functional connectivity patterns to other brain areas. Within the occipito-temporal cortex, there was increased short-range inter-regional functionally connectivity. More specifically, the left lingual gyrus of the patients had increased functional connectivity with the right ventral temporal cortex including the lateral fusiform gyrus and the parahippocampal sulcus. The right calcarine sulcus had increased functional connectivity with the right lateral occipital cortex. According to the interactive specialization view (48), such increased inter-regional functional connectivity may indicate a reduced level of functional specialization within the occipito-temporal cortex. The loci in the occipito-temporal cortex showing increased functional connectivity in the cataract-reversal patients overlap with the core areas underlying face perception (49). Reduced functional specialization in those areas may underlie the patients' behavioral deficits in face processing (32–35).

The changes of cortical thickness, cortical-thickness covariation, and functional connectivity pattern in the cataract-reversal patients are not likely to be the direct result of reduced vision in adulthood, rather than the history of early visual deprivation that led to the reduced vision. First, in the current sample of patients, the visual acuity varied in a wide range from 20/20 to 20/125 in the better eye. However, cortical thickness of the left calcarine sulcus, an area that consistently shows a difference in cortical thickness between early visual deprived patients and sighted controls, was not correlated with visual acuity. In fact, eight out of the eleven patients have acuity equal to or better than 20/50 in at least one eye, which is the minimum criterion for a Canadian driving license. Second, blind adults with onset later in childhood do not show the changes of cortical thickness reported in the early blind or found here in the cataract-reversal patients (16, 20, 21). Therefore, a history of reduced visual acuity later in life is not likely to be the cause of the changes in cortical thickness.

Unlike the early blind, who are deprived of any visual input since early childhood, the vision of the congenital cataract patients was restored within the first year of life, at the latest by 9 months of age. The timing of the restoration of visual input is well-before the age when cortical thickness has been reported to start to decrease (around 3 years) in typically sighted children (8–10, 12). Also, the occipito-temporal region appears to have a prolonged period for tuning to faces (50–57). Therefore, the role of the early visual input may function to establish the initial neural structure and processes for later changes in cortical thickness and functional connectivity based on later experience-based strengthening of neuronal connections or pruning. Such delayed effects have been referred to as a “sleeper effect” (58). Here, we demonstrated a “sleeper effect” in the development of cortical thickness and functional connectivity. In the current sample of patients, the thickness of the left calcarine sulcus was not correlated with the duration of deprivation, which varied from 2 to 9 months. In fact, there was a trend for those with shorter durations of deprivation to have thicker cortex in the left calcarine sulcus, a finding suggesting that early visual deprivation as short as 2–3 months is enough to cause long-lasting changes in cortical thickness.

In the current study, we tested only an adult sample of cataract-reversal patients at one time point. Although that allowed us to measure the long-lasting effect on cortical thickness and functional connectivity of early visual deprivation, it would be informative to test a developmental population, especially in early childhood, to record developmental changes in cortical thickness in cataract-reversal patients relative to typically developing children as they are revealed. As we show that the influence of early visual experience on brain development goes beyond the visual cortex, it would also be important to examine the association between the changes in cortical thickness and in functional brain activation and behavioral performance taking a cross-modal perspective. Such an approach will provide insight into the cross-modal deficits reported in recent studies with cataract-reversal patients (e.g., deficits in audio-visual integration) (37, 59). The small sample size in the current study, the result of the rareness of bilateral congenital cataract patients, precluded us from doing so.

In conclusion, early visual deprivation affects later interactive specialization processes of the development of cortical thickness and functional connectivity not only in the occipital lobe but throughout the brain. These changes likely reflect adverse effects on the establishment of the neural substrate for later development.

## Data Availability Statement

The datasets presented in this study can be found in online repositories. The names of the repository/repositories and accession number(s) can be found at: http://www.restfmri.net/early_visual_deprivation.zip.

## Ethics Statement

All procedures were in accordance with the 1964 Helsinki declaration and its later amendments or comparable ethical standards. The research protocols were reviewed and approved by the research ethics committees of McMaster University, The Hospital for Sick Children in Toronto, and York University. The patients/participants provided their written informed consent to participate in this study.

## Author Contributions

YF: methodology, software, formal analysis, investigation, writing—original draft preparation, writing—review and editing, and visualization, OC: conceptualization, methodology, writing—review and editing, and funding acquisition. DM: resources, methodology, and writing—review and editing. KY: writing—review and editing and funding acquisition. XG: conceptualization, methodology, software, formal analysis, investigation, data curation, visualization, funding acquisition, and supervision. All authors contributed to the article and approved the submitted version.

## Funding

This study was funded in parts by the European Research Council Starting Grant MADVIS (Grant Number 337573) (OC); Belgian Excellence of Science Program from the FWO and FRS-FNRS (Grant Number 30991544) (OC); Program of National Natural Science Foundation (Grant Numbers 81870641 and 82070939) (KY); Key Research and Development Project of Zhejiang Province (Grant Number 2020C03035) (KY); Fundamental Research Funds for the Central Universities of China (2019QN81013) (XG); Zhejiang University Global Partnership Fund (188170-11103) (XG); Zhejiang University Startup Fund (XG). OC is a research associate supported by the Fond National de la Recherche Scientifique de Belgique (FRS-FNRS). The funding sources had no involvement in the study design, data collection, data analysis, results interpretation, or writing of the paper.

## Conflict of Interest

The authors declare that the research was conducted in the absence of any commercial or financial relationships that could be construed as a potential conflict of interest.

## Publisher's Note

All claims expressed in this article are solely those of the authors and do not necessarily represent those of their affiliated organizations, or those of the publisher, the editors and the reviewers. Any product that may be evaluated in this article, or claim that may be made by its manufacturer, is not guaranteed or endorsed by the publisher.

## Footnotes

^1^Even though our sample size almost doubled (eleven vs. six) that of the previous study (41) investigating cortical thickness in congenital cataract-reversal patients, because of the rarity of such patients, we still have a relatively small sample in comparison to studies with typical adults. Therefore, we used a statistical criterion typical of previous studies of adults with abnormal early visual experience with a small sample size (e.g., uncorrected *P* < 0.001 was used in Jiang et al. comparing cortical thickness at a whole-brain level in early blind subjects with or without optic nerve damage) (16).
